# Hot-Hole Cooling Controls the Initial Ultrafast Relaxation in Methylammonium Lead Iodide Perovskite

**DOI:** 10.1038/s41598-018-26207-9

**Published:** 2018-05-25

**Authors:** Gordon J. Hedley, Claudio Quarti, Jonathon Harwell, Oleg V. Prezhdo, David Beljonne, Ifor D. W. Samuel

**Affiliations:** 1 Organic Semiconductor Centre, SUPA, School of Physics and Astronomy, University of St Andrews, North Haugh, St Andrews, Fife, KY16 9SS UK; 2 Laboratory for Chemistry of Novel Materials, Department of Chemistry, Université de Mons, Place du Parc 20, 7000 Mons, Belgium; 3 Department of Chemistry, University of Southern California, California, 90089 Los Angeles, United States

## Abstract

Understanding the initial ultrafast excited state dynamics of methylammonium lead iodide (MAPI) perovskite is of vital importance to enable its fullest utilisation in optoelectronic devices and the design of improved materials. Here we have combined advanced measurements of the ultrafast photoluminescence from MAPI films up to 0.6 eV above the relaxed excited state with cutting-edge advanced non-adiabatic quantum dynamics simulations, to provide a powerful unique insight into the earliest time behaviour in MAPI. Our joint experimental-theoretical approach highlights that the cooling of holes from deep in the valence band to the valence band edge is fast, occurring on a 100–500 fs timescale. Cooling of electrons from high in the conduction band to the conduction band edge, however, is much slower, on the order of 1–10 ps. Density of states calculations indicate that excited states with holes deep in the valence band are greatly favoured upon photoexcitation, and this matches well with the fast (100–500 fs) formation time for the relaxed excited state observed in our ultrafast PL measurements. Consequently we are able to provide a complete observation of the initial excited state evolution in this important prototypical material.

## Introduction

Methylammonium lead iodide (MAPI) perovskites and chemical analogues have seen a tremendous degree of interest, initially for use in photovoltaic cells^1–9^, and more recently also for light emitting diodes^10^ and lasers^11,12^. The key advantage of MAPI perovskite is the hybrid nature of the material, combining the benefits of conventional inorganic semiconductors such as high charge mobilities^13^ and small exciton binding energies^14^, with cost-effective solution-based deposition techniques developed for organic semiconductors^15^. While great developments have been reported in both the efficiencies of the various devices fabricated using MAPI^16^ and understanding of the underlying physics of the material^17^, significant questions remain outstanding. Key amongst these questions is what the exact nature of the initially accessed photogenerated electronic states in MAPI are, how the system relaxes to the lowest excited state, and what controls the relaxation process.

Upon absorption of a photon in MAPI, free electron-hole pairs are formed^17–20^. Recombination of these pairs can be observed with time-resolved photoluminescence measurements, deducing recombination pathways and rates on slow nanosecond timescales as the excited state depopulates back to the ground state. The very initial femtosecond processes in MAPI upon absorption of a photon have most often been measured with ultrafast transient absorption, where monitoring in the spectral region of the ground state bleach gives information on the evolution to the final excited state. Xing *et al*. followed the evolution of the photobleaching signals at 480 nm and at 760 nm for a MAPI-film in contact with a selective hole- or electron-layer, observing a strikingly asymmetric behaviour for holes and electrons^21^. An excitation dependence is reported such that if MAPI is excited at 400 nm a 400 fs rise is observed, assigned to hole relaxation from the second to first valence band (dual-valence model), while if the interband transition is excited directly from the first valence band with 600 nm excitation then no rise-time is seen. A consistent but slightly different model was proposed in another study^22^ where charge cooling takes place on a 700 fs timescale, and was assigned to both electron and hole cooling. Recently Richter *et al*. used two-dimensional photon echo experiments – a significantly more complex pump-probe-like methodology–to study carrier thermalization in MAPI, and find it to be in the region of 10–85 fs, dependent upon the excess energy of the carriers^23^. Meanwhile Ghosh *et al*. used very high time resolution pump-probe to study the earliest processes in MAPI, and find that initially created hot excitons dissociate in ~20 fs, followed by carrier thermalization on the 100 fs timescale^24^. It is clear that the ability to probe the relaxation of both charges and distinguish them is crucial to aid understanding of the fundamental processes in MAPI. The central question is: what is the nature of the ultrafast relaxation in this material? Are electrons promoted from the valence band (VB) edge to high in the conduction band (CB) before relaxing, or are they promoted from deep in the VB to the CB edge and holes “cool” to the VB edge? Crucially, as transient absorption measurements involve tracking the ground state bleach or excited state absorption bands that can shift or evolve as the excited state evolves, the ability to track the relaxation process is somewhat hampered by spectral complexity making definitive tracking of the excited state evolution *to* the final state challenging.

Observations of ultrafast photoluminescence (PL), by contrast, can greatly aid in understanding of this difficult but important problem. By measuring the ultrafast PL at different detection energies we can in effect directly monitor the excited state population at different stages in its evolution, thus a detailed picture can readily and unambiguously be constructed, from the species created upon absorption of a photon through to the final long-lived relaxed emissive state. Limited single wavelength ultrafast PL experiments in MAPI have enabled the observation of excited state relaxation times similar to those found in transient absorption to be measured^25,26^, with time constants of ~200–500 fs. However, only narrow conclusions were able to be drawn on what processes controlled this relaxation, with assignment given to equal rates of cooling of electrons to the conduction band edge and holes to the valence band edge.

Here we are able to answer the pressing question of what controls the initial relaxation in MAPI perovskite by combining a detailed and comprehensive set of observations of the ultrafast photoluminescence with an advanced theoretical treatment of the excited states using non-adiabatic quantum dynamics simulations. As mentioned above, non-relaxed excited states in MAPI can either be from within the valence band to the conduction band edge, generating hot holes, from the VB edge to higher in the CB, generating hot electrons, or a combination of both. Our PL measurements provide accurate estimations of PL rise-times at various wavelengths, enabling us to track quantitatively the photogenerated carrier dynamics. The measured PL rise-times indicate a fast relaxation of photogenerated carriers at high energy, within 100–500 fs, followed by a slower relaxation when carriers get close to the band edges, on the order of 1–10 ps. Non-adiabatic quantum dynamics simulations match the observed rise-times and enable us to assign the initial fast component of the charge relaxation to cooling of holes within the valence band and the subsequent slower component to the relaxation of the electrons within the conduction band. We find that the electron density of states strongly favours optical transitions from deeper inside the valence band to the conduction band edge, leading primarily to the generation of hot holes, and thus the overall relaxation is dominated by hot hole cooling on the 100–500 fs time range. A small amount of electron cooling to the conduction band edge also contributes, and is consistent with the significant slowing of the relaxation rate that is observed as one approaches the final lowest excited state energy, where cooling slows to ~700 fs. Overall our combined experiment-theory approach enables important new insights into the fundamental ultrafast relaxation in MAPI to be determined.

## Results

### Ultrafast photoluminescence

**Figure 1:**
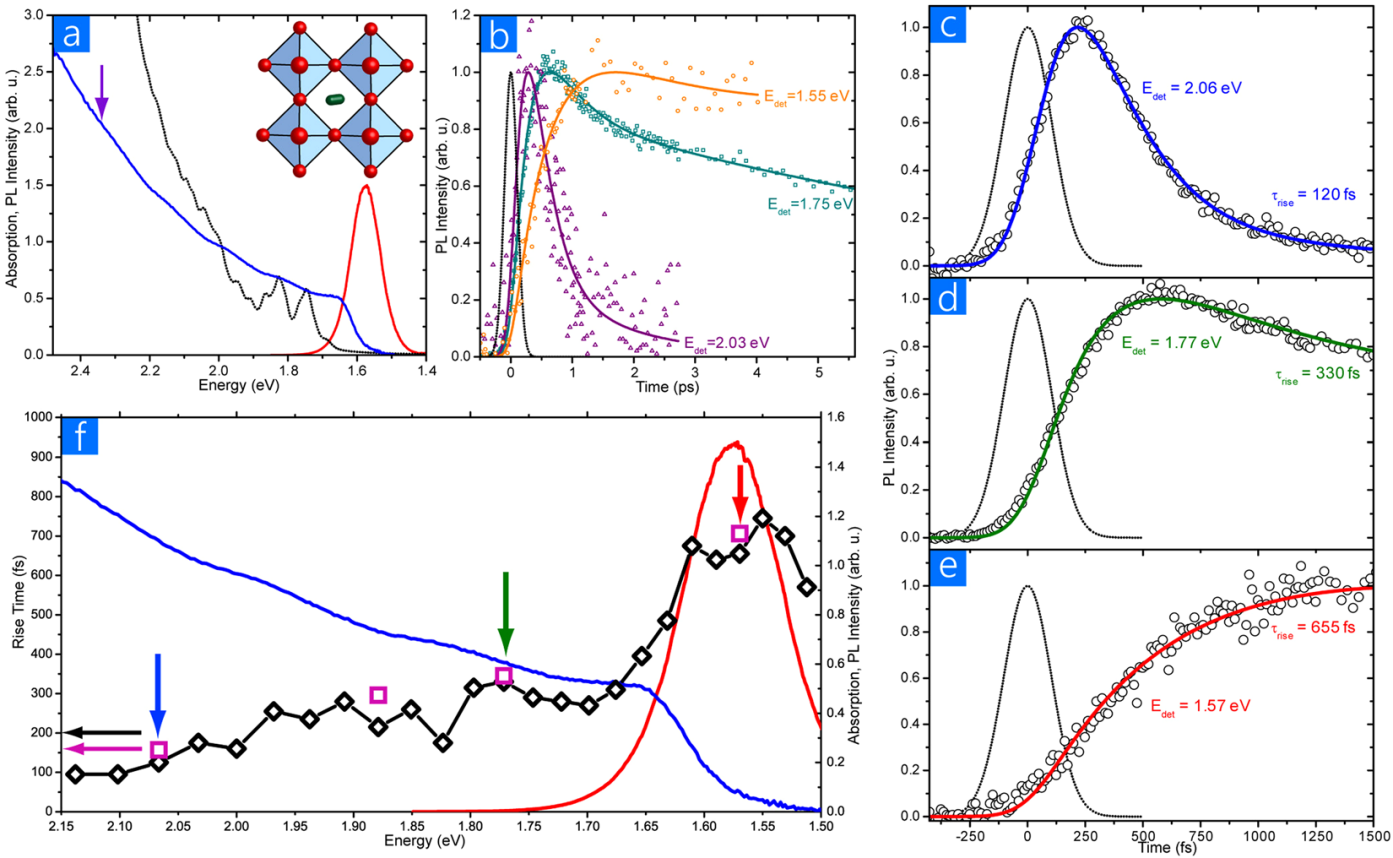
Ultrafast experimental results. (**a**) Absorption (blue line), PL (red line) spectra of methylammonium lead iodide films. The purple arrow indicates the excitation wavelength used in these studies. The black line is the theoretically calculated absorption spectrum (*vide infra*). The crystal structure is shown in the inset. (**b**) PL dynamics measured in the range 0–5 ps at three detection energies as noted. Measuring well above the bandgap leads to a fast decay, on the edge of the steady state PL a slower decay, while on the PL peak a decay so slow as to be outside the time window used here. (**c–e**) Ultrafast PL kinetics of a MAPI film in the 0–1500 fs time range at three detection energies of 2.06 eV (**c**), 1.77 eV (**d**) and 1.57 eV (**e**). The instrument response function is shown as a dotted line in each case. The solid lines represent the best-fit to the dynamics with rise-times of 120, 330 and 655 fs for each detection energy, along with subsequent fast decays as appropriate. (**f**) Fitted PL rise-times (open diamonds, left axis) as a function of detection energy. Also shown are the steady state absorption and PL spectra for reference (solid lines, right axis). The purple squares are rise-times derived from theoretical calculations (*vide infra*) and show very good agreement with experimental values. The coloured vertical arrows refer to the detection energies/rise-times shown in panels (**c–e**).

To begin our examination of the excited state processes in MAPI we look at the time-resolved photoluminescence from a thin film of the material deposited on a fused silica substrate. The steady state absorption and PL spectra of MAPI are shown in Fig. 1a along with its unit cell. MAPI perovskite shows a bandgap at 1.6 eV (775 nm), with the PL peak at 1.57 eV (790 nm). Shoulders in the absorption spectrum are evident at 680, 620 and 575 nm, possibly associated with higher energy transitions between the valence and conduction band. Steady state PL is attributed to long-lived charge recombination^17^, however the nature of any ultrafast PL is less well understood.

Ultrafast PL from MAPI films was investigated with upconversion spectroscopy. Excitation was at 2.41 eV (~0.85 eV above the PL peak), with no power dependence observed in kinetics over a factor of two of excitation fluence (see Supporting Information). An overall picture of the spectral dependence of the time-resolved PL decays is shown in Fig. 1b, where PL kinetics from energies well above the steady state PL peak, a little above the peak, and on the peak are shown out to ~5 ps. As expected, PL detected at energies above the steady state PL decay quickly as the system relaxes to the bottom of the excited state, while PL from within the steady state region decays very slowly (in the context of the time window chosen here) through radiative recombination of charge pairs back to the ground state. Noticeable in these selected time traces is that the initial formation time of PL changes as a function of the detection energy. To investigate this more carefully the rise-time region of three further energies are shown in Fig. 1c–e. It is clear that in all cases, even at a detection energy as high as 2.06 eV (600 nm), there is a delay in the formation of the PL versus what one would expect given the temporal response of the experiment. This is evidenced by the fact that the slope of the PL formation is shallower than the dotted line representing the instrument response function. The PL kinetics we would expect to measure on the instrument if the emission was instantaneous is shown in SI – indicating that the observed PL formation is demonstrably slower. Here, 350 meV of excess energy has been deposited in the perovskite, and it takes a rise-time constant of ~120 fs for the excited state to dissipate this energy and to reach the energy detection window. When one moves to detecting emission on the PL peak at 1.57 eV (790 nm, Fig. 1e) then the effect is even clearer, where 840 meV of excess energy takes a rise-time constant of 650 fs to dissipate. This is understandable in the context of relaxation of the excited state, where an effective delay time will exist at lower energy relaxed sites before the excited state reaches them. To fully quantify this effect, we have measured the PL rise-times at 20 different energies from 2.18 to 1.51 eV (570 to 820 nm in 10 nm steps). Each of these traces are fitted with an exponential rise-time constant convolved with the instrument response function and rounded to the nearest 5 fs, to enable a quantifiable parameter of the PL kinetics to be monitored as a function of detection energy/excess energy above the relaxed excited state. All these rise-time constants are plotted in Fig. 1f along with the absorption and PL spectra for reference, the fits at each individual energy are shown in SI for reference. A continual increase in the rise-time is recorded as one moves down in energy towards the steady-state PL, in line with the larger amount of energy that must be dissipated. However, the increase is not linear, and instead shows a relatively shallow gradient throughout the lower-energy part of the absorption spectrum, with a significant slowing at and beyond the absorption edge bandgap ~1.6 eV.

The measured ultrafast photoluminescence rise-times give us a powerful insight into the electronic relaxation processes occurring at the very earliest times in MAPI. It is clear from these results that such relaxations are not trivial, and are instead controlled by dissipation mechanisms within the MAPI band structure. Such a measurable relaxation contrasts with other systems, e.g. organic conjugated materials where a nearly instantaneous dissipation through high frequency vibrational modes enables the system to reach close to its final state in tens of femtoseconds. Here in MAPI even with only ~0.25 eV of excess energy to be dissipated, the excited state takes ~100 fs to do so. At the onset of the steady state PL the dissipation of excess energy slows considerably, rising to ~700 fs. As noted earlier, the indistinguishability of electron and hole cooling to the band edges makes it difficult for us to go further in our analysis of the earliest relaxations from experiments alone, thus we have performed a theoretical investigation of the ultrafast dynamics in MAPI to aid our understanding and interpretation of the experimental results.

### Theoretical simulations

To further our investigations on MAPI we have performed ground-state Born-Oppenheimer Molecular Dynamics (BOMD) to probe local distortions of the lattice, along with semi-classical Non-Adiabatic Quantum Dynamics (NAQD) simulations of the energy cooling dynamics in hot electronic states. For the latter, we resorted to the PYXAID program^27,28^ which has been successfully used by some of the authors to study the excited state properties of MAPI^29–32^. An initial excited state is defined by promoting an electron from a Kohn-Sham^33^ single-particle level in the valence band to a Kohn-Sham level in the conduction band. Subsequently, the decay of the electron/hole couple in the conduction/valence electronic states manifold is simulated by calculating the non-adiabatic couplings between single particle states and using the fewest-surface hopping algorithm to estimate the transition probabilities. The use of the Kohn-Sham single particle set does not allow electron-hole interactions to be considered. On the other hand, previous studies clearly pointed out the extremely long lifetime (of the order of tens of ns^34^) and ineffective recombination of photogenerated carriers, exceeding the Langevin limit^35^. Hence, electron-hole interaction is expected to play a negligible role in charge relaxation on the ultrafast time-scale (1.2 ps of NAQD simulations)^36^. The Kohn-Sham orbital energies and the non-adiabatic couplings were computed at the scalar relativistic PBE level of theory^37^ which is known to predict the correct band gap for MAPI perovskites^38–40^. Full details of the theoretical methods, packages used and of the computational set-up adopted are provided in the Computational Methods section. The structural model employed for the present simulations consists of a 2 × 2 × 1 supercell of the room-temperature tetragonal phase of the MAPI perovskite, shown in SI, which represents a reasonable compromise between accuracy and computational cost.

**Figure 2:**
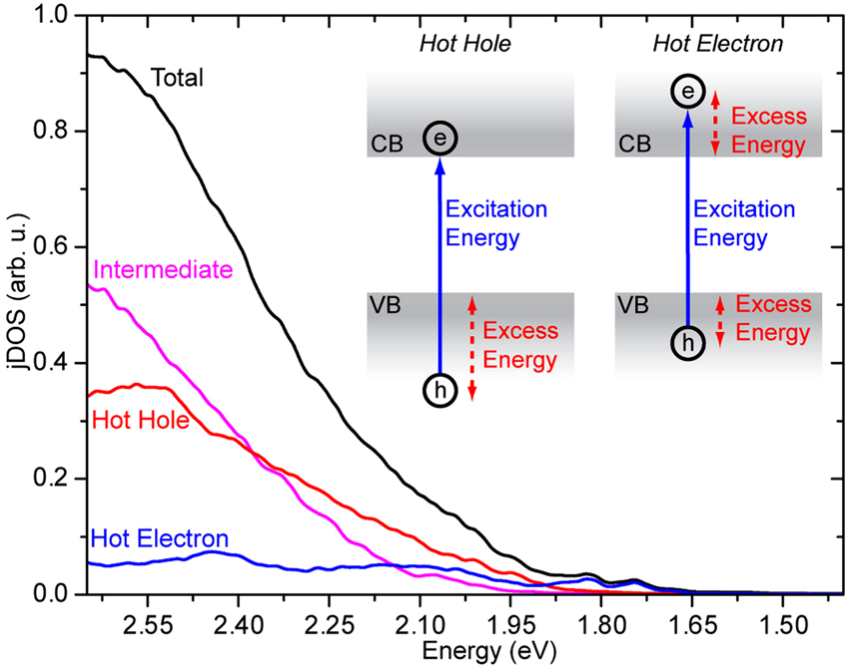
The total joint Density of States (jDOS) and jDOS associated to hot electron, intermediate and hot hole excited states. Shown in the inset is the definition we use here of the electronic excitation in terms of hot holes and hot electrons.

In our theoretical examination we first wanted to establish what the possible and likely initial configurations of the electronic transitions are. The band gap computed along the BOMD trajectory averages to 1.74 eV, in good agreement with what we observe experimentally in Fig. 1 and with similar simulations in the literature^41–44^. The oscillations of the band gap within a root mean square of 0.05 eV also agree with the same literature data. The initial electronic excited-state configuration for the NAQD simulations must be as close as possible to the PL experimental conditions. Thus we considered all the Kohn-Sham pairs separated by an energy of ~0.85 eV larger than the fundamental band gap, resulting in 68 pairs of Kohn-Sham states, from a total of 529 electronic configurations (including the ground state) in the present computational set-up. Different scenarios for these transitions exist, with the absorption of a photon resulting in the promotion of an electron either deep in the valence band to the conduction band edge (creating a “hot hole”) or from the valence band edge to a level deep in the conduction band (creating a “hot electron”), as sketched in the inset of Fig. 2. We thus classify all electron-hole pair states in these terms, with configurations where the excess of excitation energy is comparable between the two bands being classified as “intermediate”.

**Figure 3:**
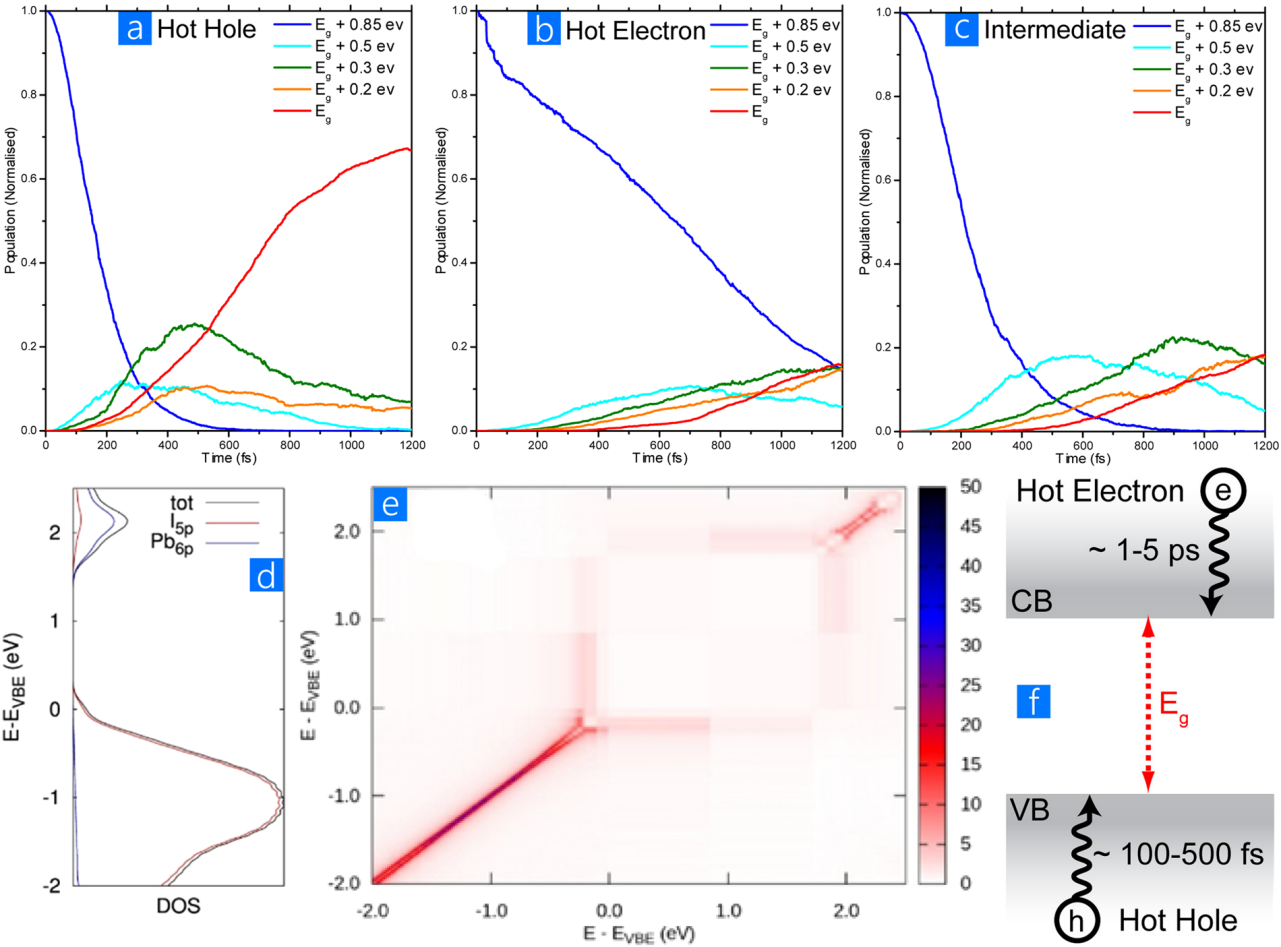
Results from NAQD simulations. (**a–c**) Time evolution of the population for excited electronic configurations over the first 1.2 ps for hot hole, hot electron and the intermediate case at energies corresponding to the band gap (E_g_) and at higher energies. The population is given as the sum of the populations of the electronic configurations at a given energy above E_g_ (within a window of ± 0.05 eV, except close to the band gap, where, we considered an energy window of 0.1 eV). (**d**) DOS computed for MAPI along the BOMD trajectory and partial DOS from 5p orbitals of iodine and 6p orbitals from lead. (**e**) Non-adiabatic couplings in the energy representation, averaged along the BOMD trajectory. The valence band edge is set to 0 eV and the couplings (z-scale) are expressed in meV. (**f**) Schematic of the observed ultrafast relaxation processes in MAPI, with holes from deep in the valence band cooling quickly, while electrons from high in the conduction band cool slowly.

In Fig. 2, we report the joint Density of States (jDOS), describing the number of electron-hole pairs at a given excitation energy. It can clearly be seen that when absorbing a photon at ~0.85 eV higher than the band gap, matching the experiments discussed above, there are many “hot-hole” and “intermediate”-like excited states available, while “hot-electron” states represent only a modest contribution (roughly 8%). This is due to the fact that MAPI has a denser DOS in the valence than in the conduction band (Fig. 3d), as widely pointed out in the literature^45,46^, which reflects the chemical composition of hybrid perovskite. Indeed, the valence band is mainly composed of 5p orbitals of the iodide, while the conduction band is mostly lead 6p orbitals, hence resulting in many more valence than conduction states, because of the 3:1 iodide:lead stoichiometry of this material. To assess the contributions from hot hole, hot electron and intermediate excitations to the optical absorption, we calculate and sum their oscillator strengths to generate a theoretical absorption spectrum, as shown in Fig. 1a. Two comments can be made, firstly that the predicted spectrum qualitatively agrees well with that measured, and secondly that the theoretical spectrum closely follows the jDOS shown in Fig. 2, demonstrating that all excitations share similar radiative rates.

That we find all transitions contributing a significant dipole moment irrespective of the starting valence and ending conduction states is intriguing and likely arises from reduced symmetry constraints in the presence of thermal lattice fluctuations in our 2 × 2 × 1 supercell model.

Having established that the ground to excited state transitions are appropriate for the MAPI system, we now turn our attention to how the excited state dynamically evolves at the early timescales after photoexcitation with NAQD simulations. Both “hot-hole”, “intermediate” and “hot-electron” states have been considered as starting configurations for the NAQD simulations, keeping in mind that in MAPI the photogenerated excited state likely corresponds to the first two types of configurations. The evolution of the electronic population for excited states at different energies above the band gap (E_g_) are summarized in Fig. 3a–c. For “hot-hole” configurations (Fig. 3a), the population of the initial high-energy (0.85 eV above E_g_) excited-state decays to ~5% within 400 fs and it goes to 0 at 600 fs. Concurrently the population of the excited states at 0.5 eV above E_g_ increases, showing a rise-time of ~160 fs, which compares well with the experimental rise time (120 fs), and then it decays. The rise time corresponds to the time required by the initial “hot-hole” configuration to cool down from 0.85 eV to 0.5 eV above E_g_. For states at 0.3, 0.2 eV above E_g_ and close to E_g_, we find rise-times of 230, 345 and 702 fs, respectively, which agree very well with the experimental values (280, 330 and 650 fs) as shown in Fig. 1c–e. The population of the electronic states close to the band gap (E_g_) goes from ~2% at 200 fs to ~52% at 800 fs and stabilizes to ~67% at 1200 fs, thus leaving ~33% of the electrons and holes in a higher energy electronic configuration.

For the initial “hot electron” configurations (Fig. 3b), however, a very different time evolution is predicted. The population of the initial, high-energy excited state decays very slowly in time and it is still considerably populated (15%) after 1200 fs. Correspondingly, the population of the excited states at 0.5, 0.3, 0.2 eV above E_g_ and close to E_g_ show only a very small increase in time. In particular, after 1200 fs, only 16% of the electrons and holes have reached the bottom of the conduction and valence band edge, respectively. Hence, in the case of initial “hot-electron” excited-states, electrons take a very long time to cool down through the conduction band. We tentatively associate this relaxation time with the measured 10 ps timescale reported by Chen *et al*.^26^. Finally, for “intermediate” initial excited-states (Fig. 3c), the population of the initial, high-energy excited states rapidly decays down to 15% of the initial value within the first 400 fs, similar to the “hot-hole” case, but the population of the states close to the E_g_ is still limited to 18% after 1200 fs, similar to the “hot-electron” case. For these electronic configurations, therefore, the holes rapidly cool to the valence band edge, but the electrons cool much more slowly from high-energy configurations, thus explaining the small population of the excited states close to E_g_ after 1200 fs.

Summarising, the presented NAQD simulations nicely reproduce the fast excited state dynamics observed in the PL measurements when starting with “hot-hole” electronic configurations, giving us a strong indication that this is the process that dominates in films of MAPI. Surprisingly we calculated a strikingly slower electron evolution in the “hot-electron” and “intermediate” cases, indicating that hot electrons cool down much slower than “hot holes” do. The observed difference in the relaxation times of electrons versus holes provides an explanation for the transient absorption measurements by Xing that showed distinctive results when a MAPI film was in contact with an electron- (PBCM) or a hole-selective layer^21^.

We are thus driven to asking the question, what is the reason for the calculated and matching experimental observations having such a significant electron-hole asymmetry? In the present formalism, the rate of the electronic transition from state *i* to state *j* depends essentially on the non-adiabatic coupling matrix element (*d*_*ij*_), i.e. large non-adiabatic coupling *d*_*ij*_, correspond to a fast *i* to *j* electronic transition. In Fig. 3e we report the energy-dependent non-adiabatic matrix elements averaged along the BOMD trajectory. Firstly, we note that (as anticipated) the only non-zero coupling values are close to the diagonal, meaning that the non-adiabatic couplings are large only for Kohn-Sham states close in energy. Secondly, non-adiabatic couplings are larger in the valence band (with values up to 50 meV) than in the conduction band (~10 meV). Thirdly, there is a clear correspondence between the DOS and the value of the non-adiabatic coupling, that is, non-adiabatic couplings are large in the energy range of the maximum of the DOS (~1 eV below the valence band and 0.5 eV above the conduction band), while they decrease in correspondence of the band edges where the DOS is small. This is clearly explained by the fact that a high density of states ensures close energy separation between coupled electronic states and efficient release of the excess energy to nuclear degrees of freedom, which is reflected in a larger value of the NA couplings. Thus, the larger non-adiabatic couplings for states within the valence band compared to the conduction band follow the shape of the corresponding DOS. The conjunction of the larger non-adiabatic couplings and the higher density of available states lead to holes relaxing faster than electrons and to hot-electrons relaxing slower close to the band edge. These conclusions allow the two different gradient regimes of rise-times in the experimental results (Fig. 1f) to be fully understood. It is worth stressing that the present argument, which correlates fast/slow non radiative recombination to a larger/smaller density of states, represents a different mechanism from the proposed “phonon bottleneck”, to explain the slowing of the hot-charge relaxation close to the band edge^47,48^. Recent work has in fact observed a similarly slow hot-charge relaxation, even under low fluence, where the energy of the hot-carrier is expected to be effectively dissipated by phonons^49^. We can extend our analysis to investigating the phonons relevant for the electronic processes, by taking a Fourier transform of the time-dependent band gap autocorrelation function, as shown in SI. Large components in the frequency region below 100 cm^−1^ are calculated, assigned mainly to the vibration of the inorganic framework^50^; there is also a component at 115 cm^−1^, which matches the measured Raman signals found at 109 and at 119 cm^−1^ in thin-films and mesostructured samples, respectively^51^.

## Discussion

In this work we have combined ultrafast photoluminescence measurements with Non-Adiabatic Quantum Dynamics (NAQD) simulations to gain a detailed insight into the initial excited state processes in MAPI perovskites. By observing the femtosecond photoluminescence we are directly measuring the dynamics of the emissive species photogenerated in the sample, free from any complications of assignment that can make other techniques such as pump-probe spectroscopy challenging. The experimental results are interpreted on the basis of NAQD simulations, which follow the time evolution of the initial excited state, allowing us to present an overall picture of the earliest electronic processes.

Measuring the observed rise-times in the PL allows assessing the processes that control the delay in the excited state reaching the detection energy. Ultrafast relaxation, from the initial high-energy state populated with laser excitation to the final relaxed excited state, from which slower radiative and non-radiative decay occurs, can vary depending upon the system. In organic materials this initial relaxation is very fast, typically <100 fs^52^, for internal conversion between electronic states or even for vibrational redistribution amongst high frequency modes^53^. Slower picosecond relaxations, typically induced by coupling with low frequency vibrational modes, e.g. torsional rotations, also occur in organic molecules, but generally do so from the already populated lowest electronic excited state and thus lead to only a small relaxation and spectral evolution^54^. In direct bandgap inorganic semiconductors, instead, relaxation of electrons and holes to the band edge can be considerably slower, in the picosecond time range, and are mediated by lattice phonon modes. Our joint experimental-theoretical study points to a distinctive behaviour occurring in MAPI perovskite. Ultrafast PL measurements performed for optical excitation 0.85 eV above the PL peak indicate relaxation processes in the sub-picosecond time scale, with fast relaxation when significantly above the relaxed excited state, but slowing considerably when it nears it. Well above the electronic band gap, the rise-times are of the order of 100–300 fs, while close to the band gap, the rise-times are longer, in the range of 300–700 fs (Fig. 1f). Given that such dynamics represents the formation time of emissive states, the general interpretation is that optical absorption generates electron/hole pairs that quickly dissociate and relax before contributing to non-geminate recombination and photon emission on longer timescales^17^. The theoretical calculations enable us to derive more exacting conclusions from the experimental results, show that above bandgap light absorption in MAPI likely results in promoting an electron from a deep valence band level to the conduction band edge (so-called “hot-hole” excited states), and predict rise-times for these “hot hole” like excitations in very good agreement with our experimental results. Thus, the theoretical calculations support the view that the PL measurements reported here probe relaxation of hot-holes in the valence band and provide theoretical support to the dual-valence model^21^. Moreover, theoretical simulations also predict that the electron excitation from the valence band edge to a high-energy conduction band level (a “hot-electron” state) is less likely and characterized by much longer lifetimes, on the order of ps^26^. This is also consistent with the long lifetime (10^2^ ps) for hot-carriers recently reported for CH_3_NH_3_PbBr_3_ perovskites^49^. A recent publication by Madjet *et al*., appeared during the preparation of this manuscript, also underlying an asymmetric behaviour for electron and hole relaxation^36^. However, these authors do not discuss whether the initial photogenerated species corresponds to “hot-hole” rather than to “hot-electron” like excitation, which is the key result of the present work, and leads to a fuller understanding of the optoelectronic properties of hybrid perovskites^34^. The ability to discriminate between charges in the very earliest time evolution of the MAPI excited state that we report here is of great value, as this is very difficult to achieve given the relative indistinguishability of electrons and holes. Furthermore these results reach the earliest possible times after photoexcitation of the material, and tell us about the nature of the energy landscape that both charges experience as they relax.

These findings are important, as they help to elucidate the nature, timescales and details of the very fastest initial processes in MAPI perovskite. Understanding of such details is important if one wants to think about taking advantage of the unique properties of these materials. For example, such new knowledge may open up possible strategies for hot-carrier extraction that would take advantage of the long lifetime of “hot-electron”-like states that we find here. Similarly, perovskite-based lasers could benefit from the long life times of these “hot-electrons”, which are crucial for the population inversion process.

## Experimental Methods

### Film preparation

Perovskite films were prepared by the lead acetate precursor route described by Zhang *et al*.^55^. A solution of 221 mg of recrystallised methylammonium iodide (MAI) and 175.6 mg of lead acetate dissolved in 1 ml of N-N dimethylformamide (DMF) and stirred for 5 minutes until the solution had completely dissolved. Substrates to be spin coated were first cleaned via sequential sonication in Helmanex solution, deionised water, acetone, and then isopropanol before being dried and then oxygen plasma cleaned for 3 minutes at maximum power. The solution was then spin coated onto the clean substrate in a nitrogen filled glovebox (<0.1ppm O_2_ and H_2_O) at 2000 RPM for 60 s, followed by vacuum annealing at 100 °C for 5 minutes. Samples were ~120 nm thick with <10 nm roughness, and any samples with PbI_2_ impurities (shown by a “milky” appearance on the surface of the film) were discarded. Samples were encapsulated by placing a 2 mm thick fused silica window on top of the film and sealing it in a rotating sample holder for measurement in the upconversion setup.

### Ultrafast photoluminescence

Ultrafast PL was measured with a modified FOG100 upconversion setup by CDP Systems. Briefly, the 1030 nm, 80 MHz, 100 fs fundamental of an Yb:KGW oscillator (FLINT by Light Conversion) is fed into the setup, where the second harmonic at 515 nm is generated with a BBO crystal. This forms the excitation beam that is passed through a Berek polariser set to magic angle with respect to the detection polarisation, then focussed onto the sample, which was rotated to avoid sample degradation, while PL was collected with a short focal length lens. The residual 1030 nm fundamental that was not converted for the excitation was instead sent down an optical delay line and then focussed onto a BBO upconversion crystal along where it was spatially overlapped with the collected PL. Upconverted photons in the visible or near-UV were then filtered spectrally and spatially before being detected with a photomultiplier tube. Computer control of the delay line along with PMT counting enable the PL intensity as a function of time to be recorded. The instrument response function was recorded by measuring the water Raman shift of the excitation laser. The optical path length was small (~0.5 mm) to ensure dispersion did not broaden the response significantly compared to the thin perovskite film. This gave a Gaussian response of width 244 fs full-width half-maximum.

## Computational Methods

### Electronic structure calculations

Electronic structure calculations are performed at the DFT level of theory, within the pseudopotential/plane-wave implementation of the PWSCF program from the Quantum-Espresso suite^56^. We resort to ultrasoft, scalar-relativistic pseudotential, along with PBE functional to describe exchange-correlation^37^, and we used a cutoff of 25 Ry and 200 Ry, respectively for the expansion of the wavefunction and electron density. The present computational set-up has been largely exploited in the literature and represents the best compromise between accuracy and computational cost^41–43^.

For the 2 × 2 × 1 supercell model of the tetragonal phase, we employed the cell parameters by Poglitsch and Weber (total cell parameters of 17.72 × 17.72 × 12.67 Å)^57^. During the BOMD simulations, we resort to a Monkhorst-Pack mesh^58^ in the first Brillouin zone of 1 × 1 × 2, to preserve a comparable accuracy of the atomic forces along the three crystallographic directions, in line with similar calculations reported in the literature^41,42^. During the evaluation of the non-adiabatic matrix elements, instead, we considered only Γ. The comparison of the DOS for the supercell model evaluated with the two sets of k-point mesh shows that there are only minor differences between the two cases and with respect to a reference calculation of the tetragonal unit cell, using a 4 × 4 × 4 sampling of the first Brillouin zone (see Supporting Information).

### Born-Oppenheimer molecular dynamics (BOMD)

BOMD is conducted under NVT conditions, for a total simulation length of 4.5 ps. This includes 1.5 ps of production dynamics, performed after 3 ps of thermalization, where the target temperature (300 K) was reached following three steps of 1 ps duration each, with progressive increase of the temperature by 100 K. The procedure is similar to the one reported in ref.^42^, with the use of ~1 fs time steps, along with the Anderson thermostat to control the temperature, with a time constant of ~5 fs, in line with other BOMD simulations reported in the literature^34,44,59^.

### Non-adiabatic quantum dynamics

We carried out NAQD simulations using the PYXAID program^27,28^. Out of the 68 electronic excited state configurations having similar energy to the initial excitation used in PL measurements (0.85 eV above E_g_), we selected four “hot-hole”, four “intermediate” and four “hot-electron” as initial excited states configurations for the NAQD simulations, along with ten different initial conditions. The electronic configurations were then allowed to evolve in the NAQD simulations, using the Fewest Surface Hopping algorithm, along the 529 electronic configurations resulting from the current computational set-up, considering 300 stochastic realizations for each initial condition^34^.

No decoherence effects were considered in the present NAQD simulations. The need for a decoherence correction to a quantum-classical NA dynamics scheme is judged by comparing the decoherence timescale with the timescales of quantum transitions between electronic levels. In the case of intraband relaxation, investigated here, transitions happen within a dense manifold of electronic states that have similar properties^60^. Because the gaps between electronic states are small, approaching 0, transitions are very fast. On the other hand, the electronic states have similar properties, including potential energy surfaces, hence resulting in slow decoherence. As a result, the decoherence timescale is longer than quantum transition time, and a decoherence correction has little or no effect on the dynamics^35^.

### Data Availability

The research data supporting this publication can be accessed at 10.17630/6f533425-f4b6-49c9-aa69-ea02692c6af0.

## Electronic supplementary material

Supplementary Information
