# Supplementary material for: Hot-Hole Cooling Controls the Initial Ultrafast Relaxation in Methylammonium Lead Iodide Perovskite
Source: Sci Rep. 2018 May 25;8:8115. doi: 10.1038/s41598-018-26207-9 (PMC5970208; doi:10.1038/s41598-018-26207-9)
Supplement: Supplementary file 1 — Supplementary Information [file 41598_2018_26207_MOESM1_ESM.pdf]

Hot-Hole Cooling Controls the Initial Ultrafast Relaxation in Methyl Ammonium Lead Iodide Perovskite

Gordon J. Hedley,[1†] Claudio Quarti,[2†] Jonathon Harwell,[1] Oleg V. Prezhdo,[3] David Beljonne[2] and Ifor D.W. Samuel[1]

[1] Organic Semiconductor Centre, SUPA, School of Physics and Astronomy, University of St Andrews, North Haugh, St Andrews, Fife KY16 9SS, UK

[2] Laboratory for Chemistry of Novel Materials, Department of Chemistry, Université de Mons, Place du Parc 20, 7000 Mons, Belgium

[3] Department of Chemistry, University of Southern California, California 90089, Los Angeles, United States

† These authors contributed equally to the work

Supporting Information

Power Independence of Kinetics

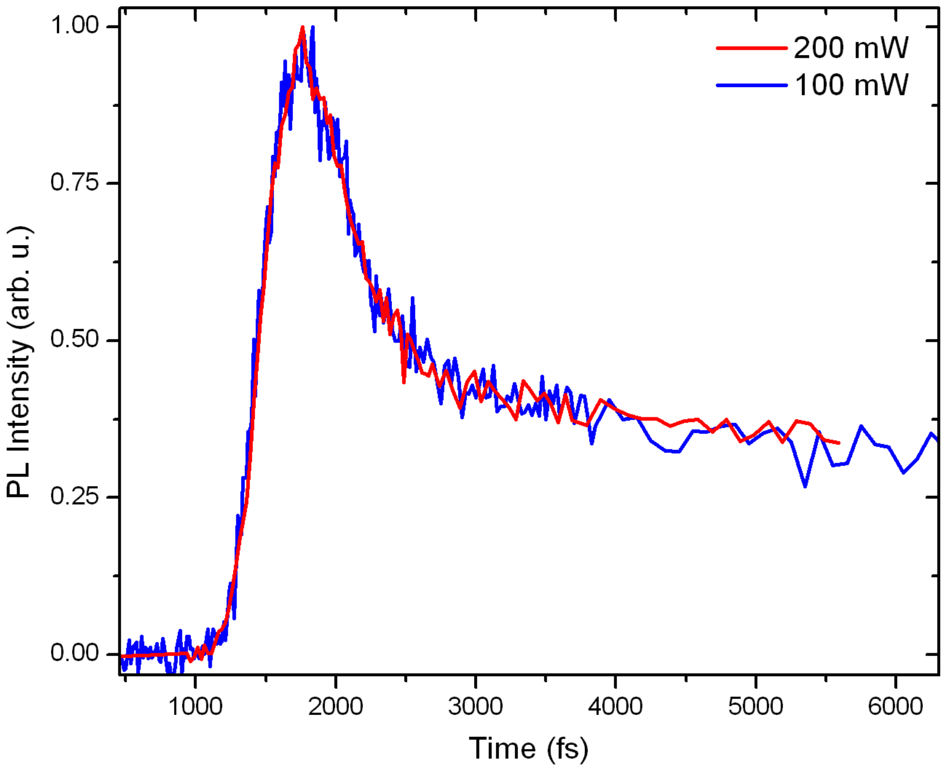

Figure S1: Ultrafast PL detected at 690 nm with excitation power reduction by a factor of two leading to no difference in kinetics.

Comparison of a Measured PL Rise-Time with an Instantaneous Rise

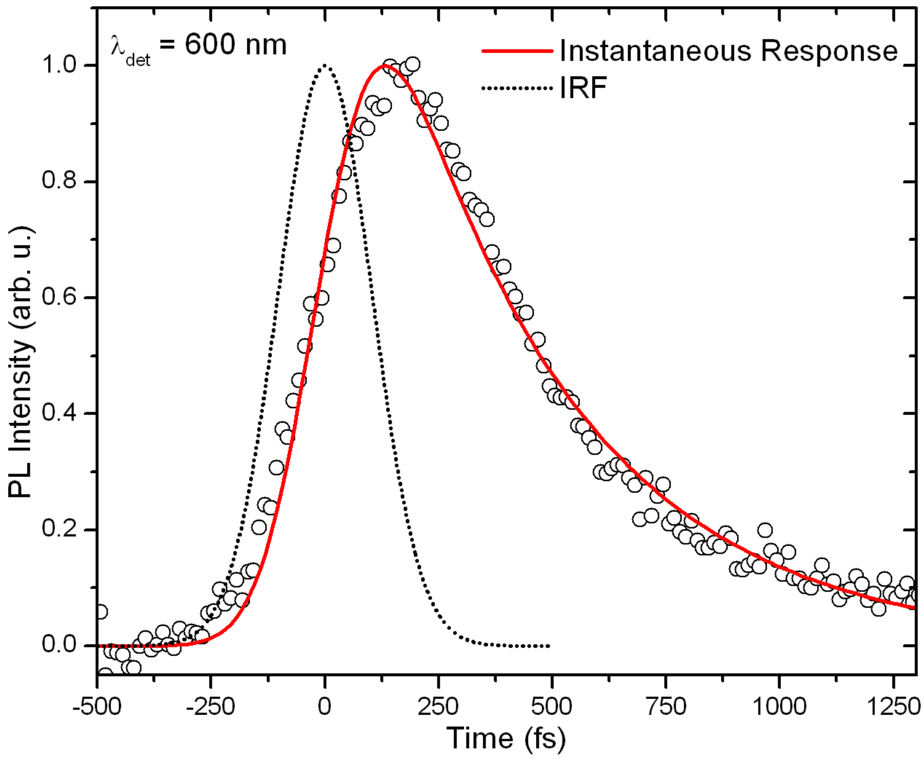

Figure S2: Ultrafast PL measured at 600 nm, with an instantaneous rise-time shown as a red line displaying a poor fit on the rise. The rise is zoomed in Figure S3. The instrument response function is shown as the dotted line.

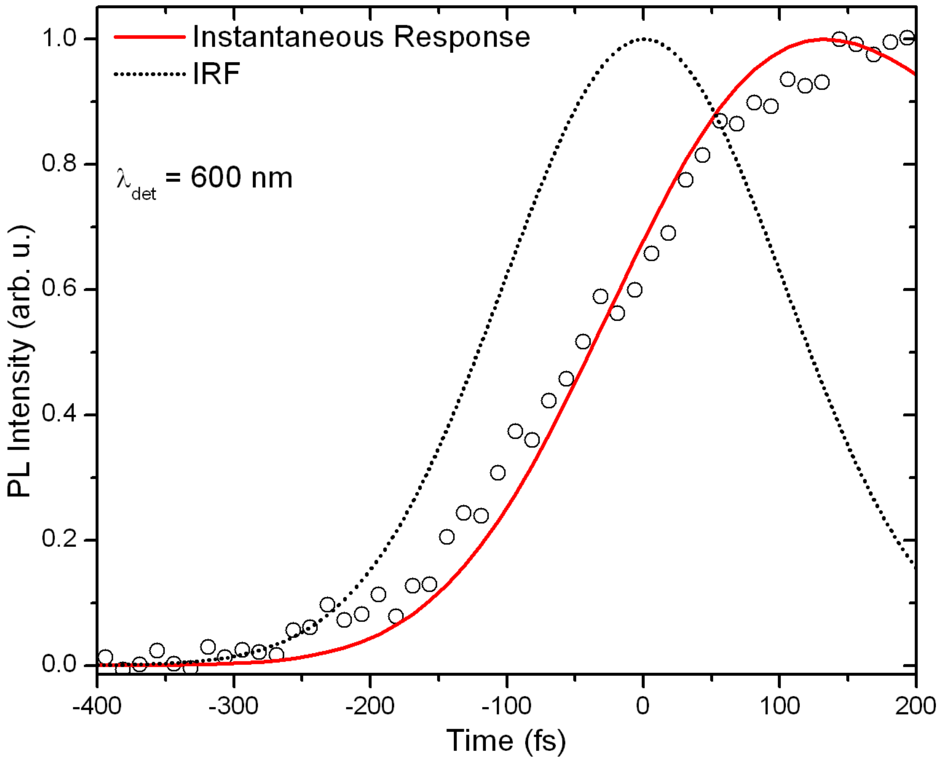

Figure S3: Ultrafast PL measured at 600 nm, with an instantaneous rise-time shown as a red line displaying a poor fit on the rise. The instrument response function is shown as the dotted line.

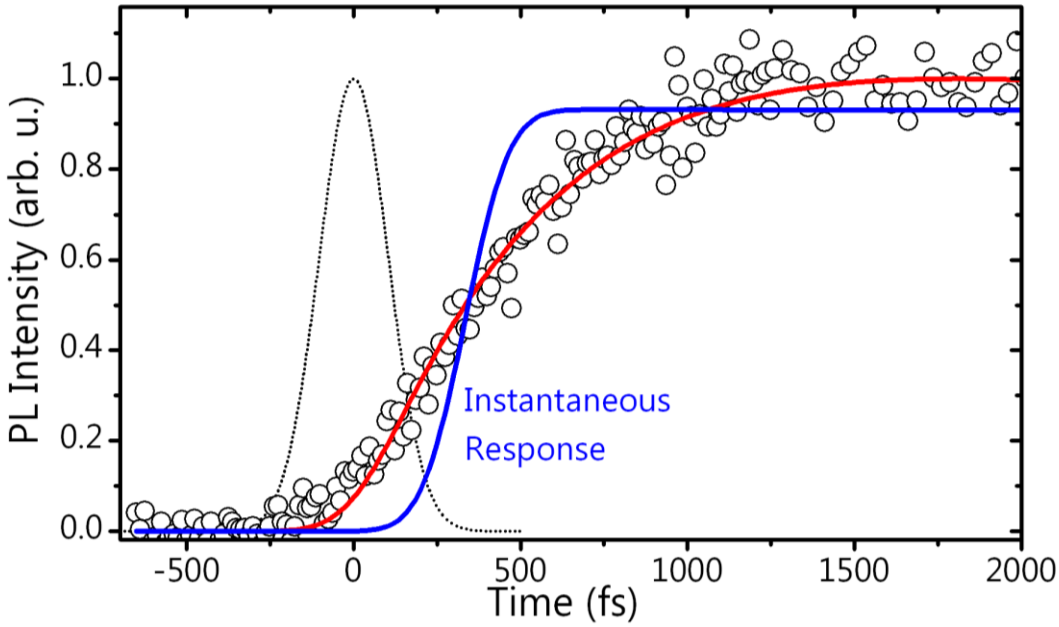

Figure S4: Ultrafast PL measured at 790 nm, with an instantaneous rise-time shown as a blue line displaying a poor fit on the rise. The actual rise-time fit of 655 fs is shown as a red line. The instrument response function is shown as the dotted line.

Wavelength Dependent Ultrafast PL Kinetics

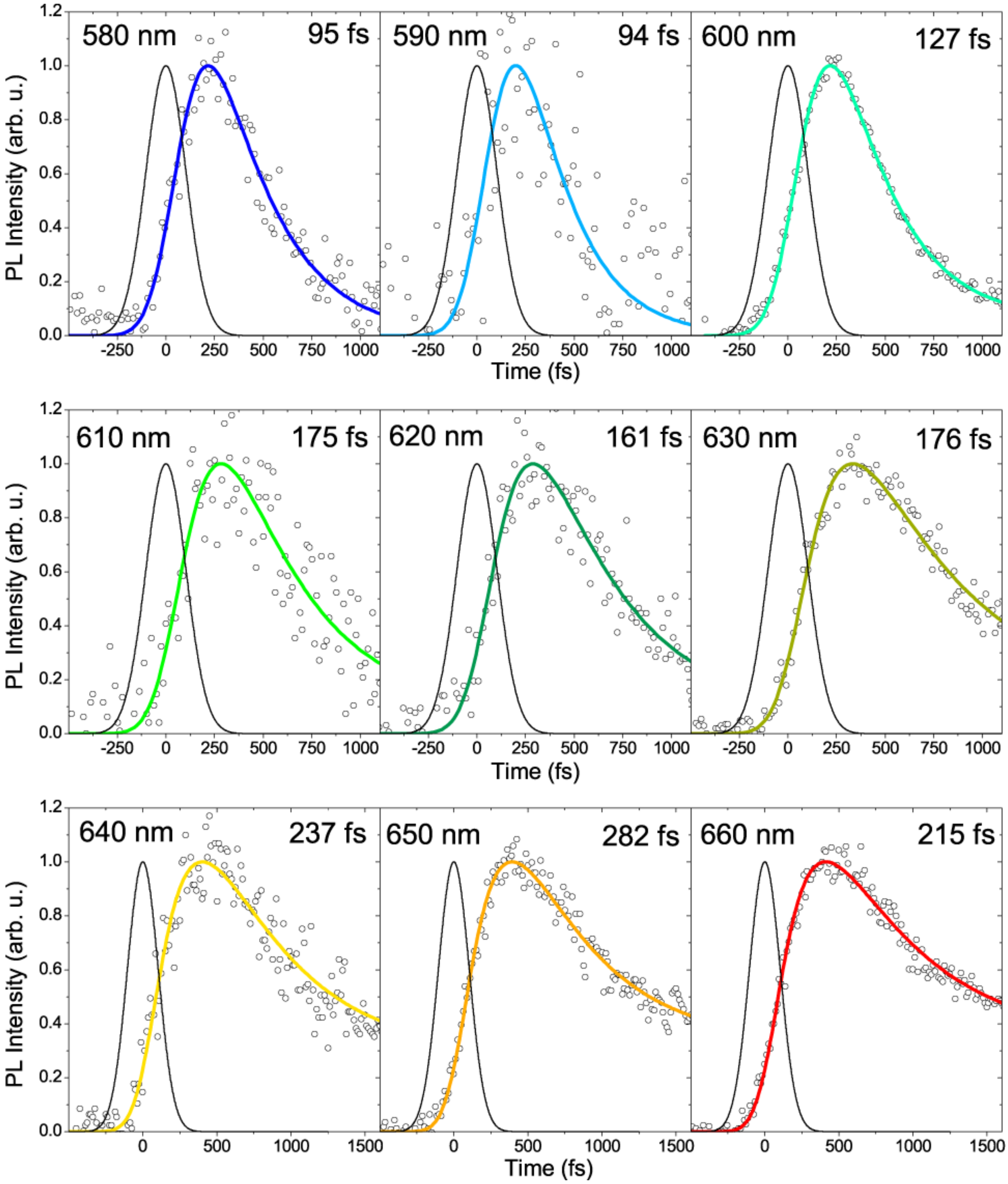

Figure S5: Ultrafast PL decays at individual wavelengths in MAPI, with the experimental conditions as denoted in the methods section. The detection wavelength is as shown in the top left of each panel, and the fitted rise-time constant as shown in the top right. The instrument response function is shown as the solid black line, the measured data points as open circles and the fit as the solid coloured line.

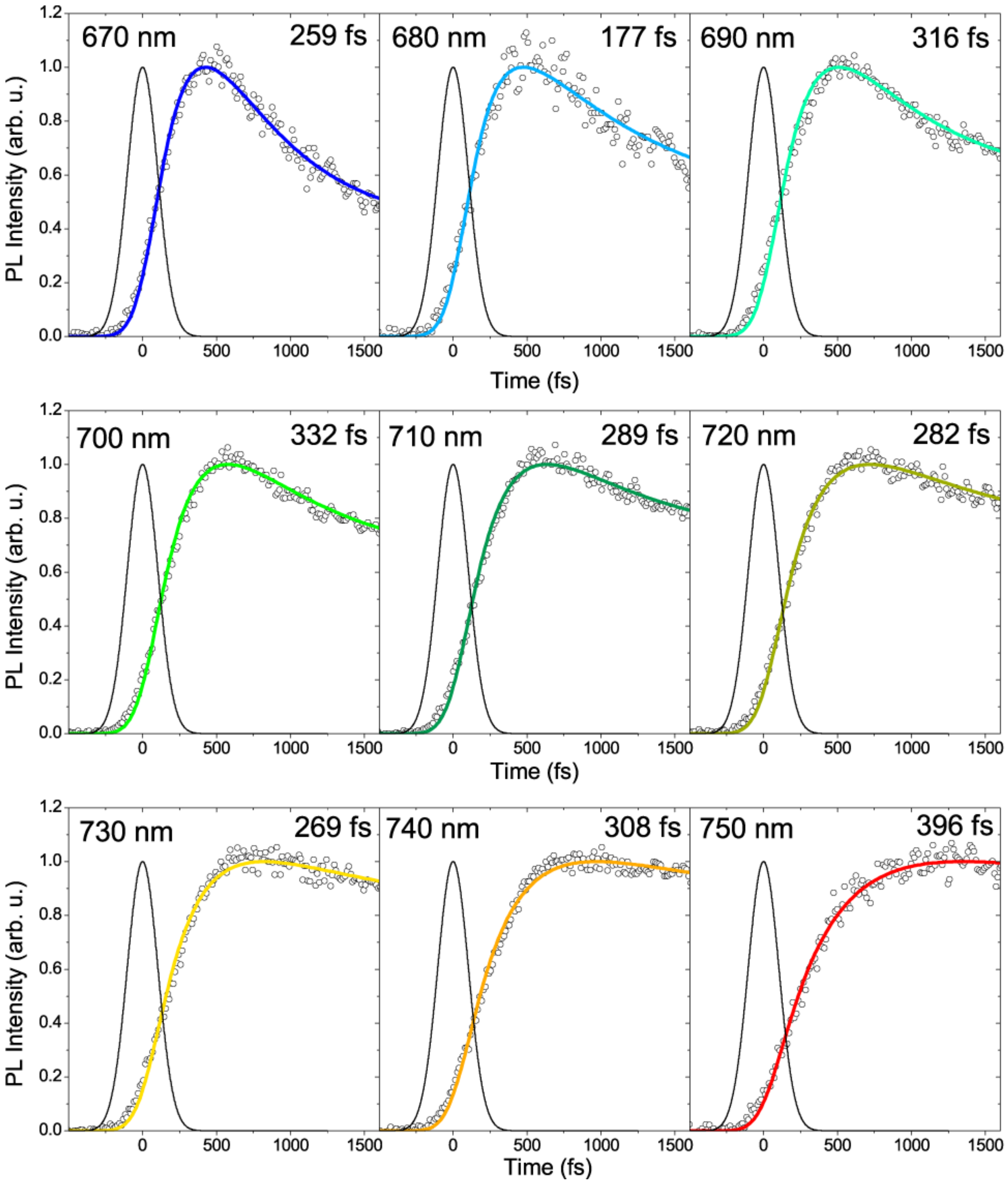

Figure S6: Ultrafast PL decays at individual wavelengths in MAPI, with the experimental conditions as denoted in the methods section. The detection wavelength is as shown in the top left of each panel, and the fitted rise-time constant as shown in the top right. The instrument response function is shown as the solid black line, the measured data points as open circles and the fit as the solid coloured line.

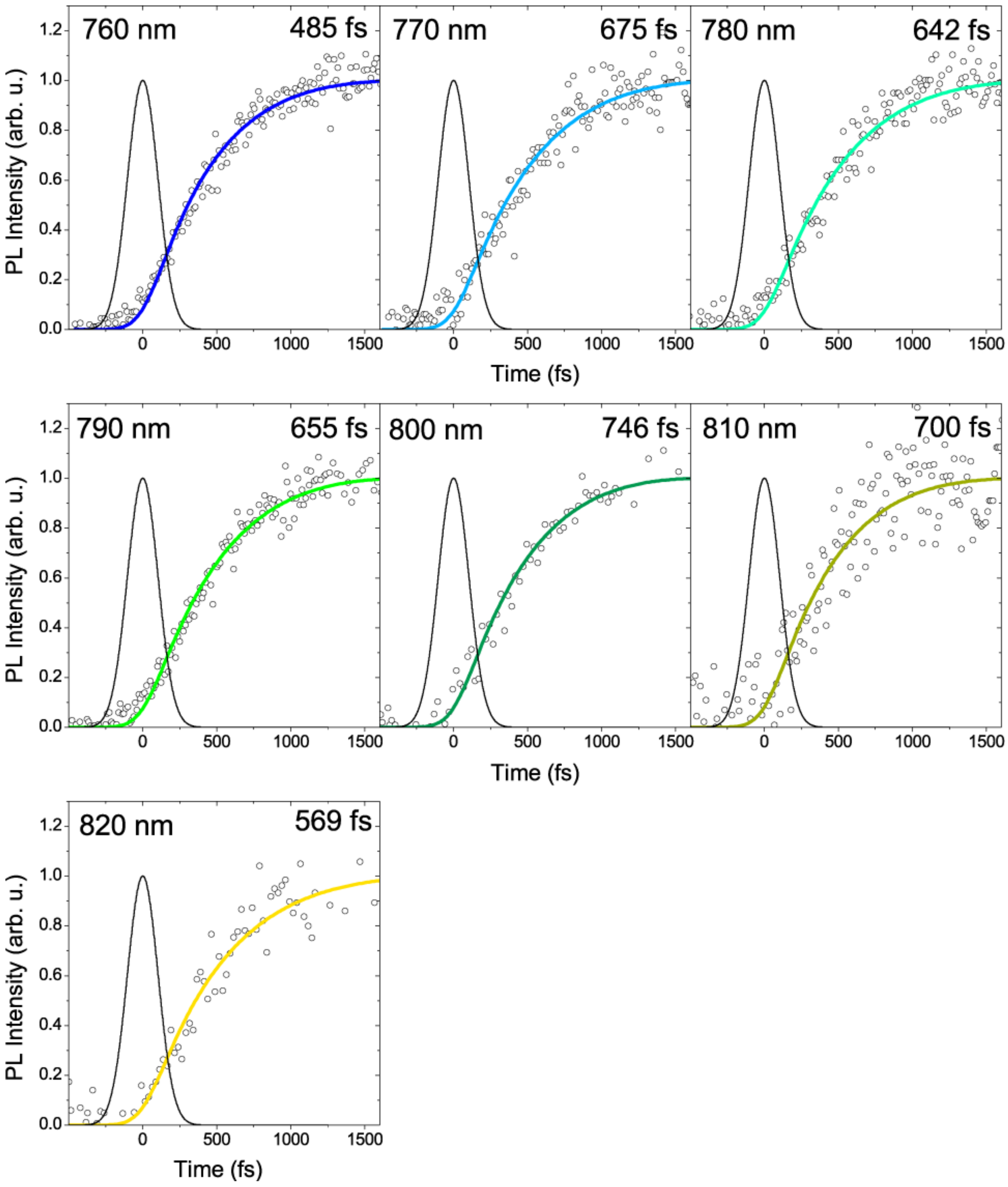

Figure S7: Ultrafast PL decays at individual wavelengths in MAPI, with the experimental conditions as denoted in the methods section. The detection wavelength is as shown in the top left of each panel, and the fitted rise-time constant as shown in the top right. The instrument response function is shown as the solid black line, the measured data points as open circles and the fit as the solid coloured line.

MAPI structural model for BOMD and NAQD calculations

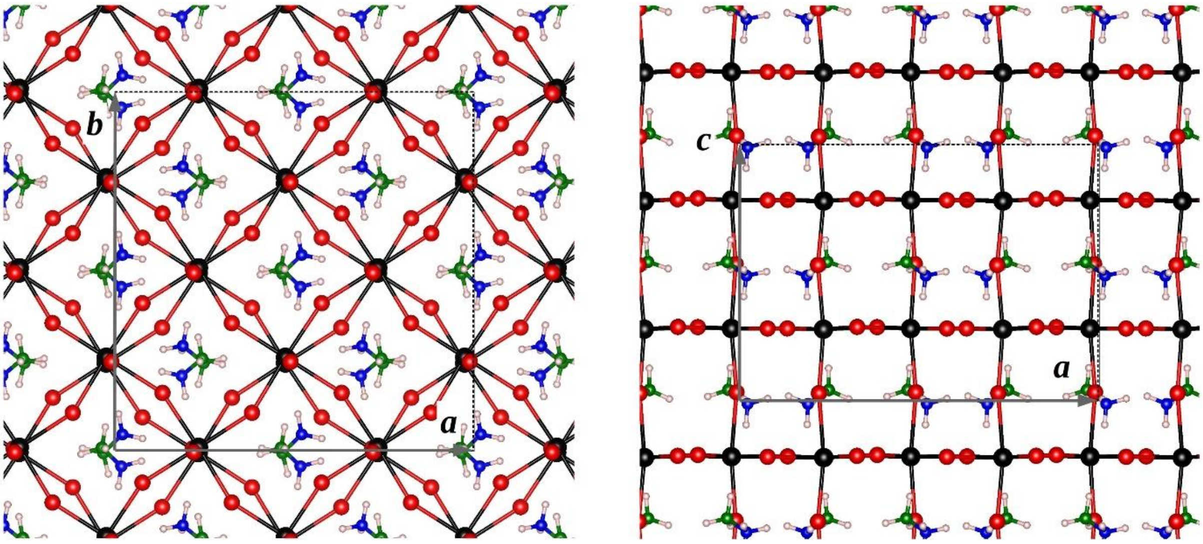

Figure S8: 2x2x1 supercell structural model of the room-temperature tetragonal phase of MAPI, employed for the present theoretical simulations. The unix cell and the cell parameters are depicted. The color scale is: lead=black, iodine=red, carbon=green, nitrogen=blue and hydrogen=white.

Fourier Transform of the Time-Dependent Band Gap Autocorrelation Function

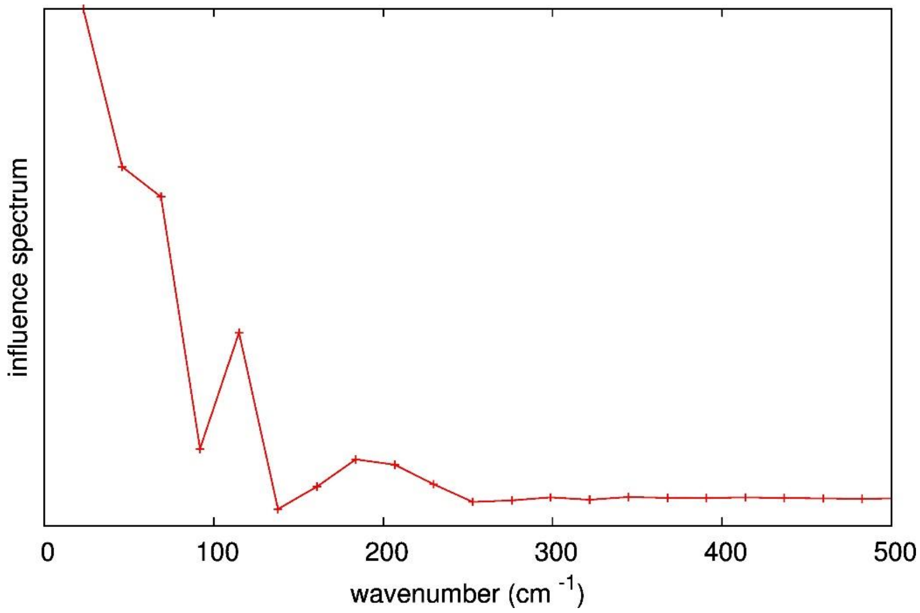

Figure S9: Fourier transform of the time-dependent band-gap autocorrelation function obtained from the present theoretical simulations. The peaks in the present function are informative of the phonons frequencies that are mainly related to the time-dependent band-gap oscillations.

4x4x4 Sampling of the First Brillouin Zone

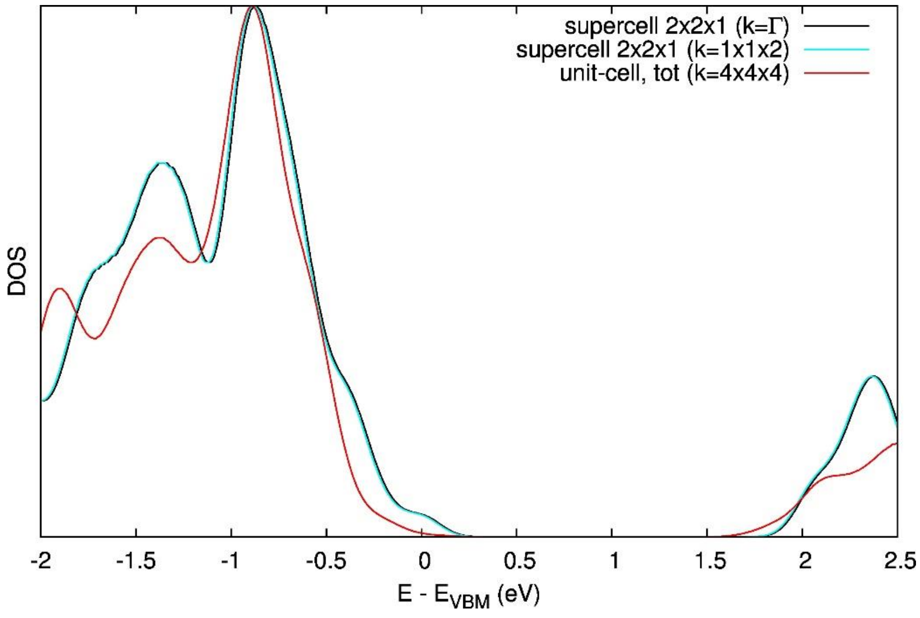

Figure S10: Comparison of the Density of States for the models and computational set-up used in the present theoretical simulations (supercell 2x2x1 with k-point sampling at Γ at k=1x1x2, respectively for the NAQD and BOMD calculations) compared with a standard DFT model, composed by the unit cell and a k-point mesh of 4x4x4.
